# Traditional Chinese Medicine Shen-Yuan-Dan (SYD) Improves Hypoxia-Induced Cardiomyocyte Apoptosis in Neonatal Rats by Upregulating miR-24/Bim Pathway

**DOI:** 10.1155/2022/5804187

**Published:** 2022-02-03

**Authors:** Fuyong Chu, Xue Yan, Xingjiang Xiong, Mingxue Zhou, Yupei Tan, Yixuan Li, Wei Liu, Hongxu Liu

**Affiliations:** ^1^Department of Cardiology, Beijing Hospital of Traditional Chinese Medicine, Capital Medical University, Beijing 100010, China; ^2^Department of Psychology and Sleep Medicine, Guang'anmen Hospital, China Academy of Chinese Medical Sciences, Beijing 100053, China; ^3^Department of Cardiology, Guang'anmen Hospital, China Academy of Chinese Medical Sciences, Beijing 100053, China; ^4^Beijing Hospital of Traditional Chinese Medicine, Capital Medical University, Beijing Institute of Traditional Chinese Medicine, Beijing, China; ^5^Department of Traditional Chinese Medicine, Community Healthcare Center of Shangzhuang Town, Haidian District, Beijing 100094, China

## Abstract

*Background*: Acute myocardial infarction (AMI) is the leading cause of malignant arrhythmia, heart failure, and sudden death. However, safe and effective drugs for the treatment of AMI are unavailable to date. The present study aimed to investigate the role of traditional Chinese medicine shen-yuan-dan (SYD) in hypoxia-induced cardiomyocyte apoptosis in neonatal rats. In addition, the study explored the possible mechanism through which SYD could reduce myocardial ischemia apoptosis and regulate the expression of the miR-24/Bim pathway. *Methods*: Hypoxia-induced neonatal rat cardiomyocytes were used for the experiments. These cardiomyocytes were transfected with an miR-24 mimic and an miR-24 inhibitor and then cocultured with SYD-containing serum. MTT and lactate dehydrogenase (LDH) assays, AnnexinV/PI double staining, flow cytometry, and TUNEL staining were used to determine the cell viability and apoptosis under hypoxic conditions. Furthermore, the expression level of Bim in the hypoxia-induced cardiomyocytes was determined through western blotting and quantitative real-time polymerase chain reaction. *Results*: After 48 h of hypoxia, LDH and creatine phosphokinase (CPK) activities increased, cell viability decreased, and miR-24 expression upregulated in the cardiomyocytes. SYD alleviated hypoxia-induced cardiomyocyte injury, decreased LDH and CPK activities, increased cell viability, and reduced apoptosis in the neonatal rat cardiomyocytes. Moreover, SYD could upregulate miR-24 expression and downregulate Bim expression. Upregulation of miR-24 expression significantly enhanced the effect of SYD, thereby improving myocardial cell apoptosis. Dual-luciferase reporter assay and western blot analysis confirmed that Bim was a direct target of miR-24. *Conclusion*: SYD treatment reduces hypoxia-induced myocardial apoptosis by upregulating miR-24 expression. This study provides new insights into the molecular mechanism underlying the therapeutic potential of SYD in promoting the recovery of myocardial function and delaying the incidence of heart failure.

## 1. Introduction

Acute myocardial infarction (AMI) corresponds to acute ischemic myocardial necrosis [1]. It is a severe coronary heart disease, which is characterized by a drastic reduction or interruption in blood supply to the coronary artery, resulting in severe and long-term ischemia of the corresponding myocardium, culminating in myocardial necrosis. AMI is also characterized by acute onset and high mortality. Additionally, it is the leading cause of malignant arrhythmia, heart failure, and sudden death. Studies have indicated that approximately 3.5 million people die from cardiovascular diseases annually in China, of which the number of myocardial infarction (MI) cases is approximately 1 million [2]. AMI has become the leading cause of death in China.

Apoptosis is a vital pathological mechanism of early cell injury after MI [3]. Extensive cardiomyocyte apoptosis can be observed during both sustained myocardial ischemia after infarction and the ischemia-reperfusion period, and the increased apoptosis of cardiomyocyte eventually promotes the occurrence of heart failure. Therefore, for terminally differentiated cardiomyocytes lacking the regenerative capacity, developing strategies to decrease apoptosis of myocardial cells after MI is crucial for promoting the recovery of myocardial function and delaying the heart failure incidence after MI.

Previous studies suggest that the Bcl-2 homology domain 3 (BH3)-only pro-protein, namely Bim, is the pivotal regulatory protein in apoptosis, which can suppress pro-survival proteins or activate pro-apoptotic proteins to initiate the apoptosis pathway in response to adverse stimuli [4, 5]. Moreover, an in vitro study has revealed that hypoxia with low glucose can distinctly promote Bim expression and induce cardiomyocyte apoptosis [6]. Bim1 triggers apoptosis by inhibiting the Bcl-2 anti-apoptotic function and/or causing direct activation of Bax, leading to cytochrome c release and apoptotic signaling pathway activation [7].

MicroRNAs (miRNAs or miRs), approximately 18–24 nucleotides in length, belong to a class of evolutionarily conserved endogenous noncoding single-stranded small RNA molecules that regulate gene expression by binding to the 3′ UTR within target mRNAs to inhibit translation or degrade target messenger RNAs [8]. miR-24 is expressed highly in various tissues and is involved in the occurrence and development of several diseases, such as heart failure, oral cancer, hyperglycemia, and Alzheimer's disease [9]. Studies have shown that miR-24 is highly expressed in the mammalian myocardial tissues and is significantly upregulated in the early stage of myocardial ischemia in mice. It also participates in the self-repair process of ischemic myocardium by regulating the activation of its target gene Bim and its downstream mitochondrial apoptotic signaling pathway [10, 11]. In addition, our previous clinical study demonstrated a significant negative correlation of miR-24 expression in peripheral blood of patients having acute coronary syndrome with the inflammatory and apoptotic factors [12]. Thus, we assume that miR-24 is closely related to coronary heart disease (CHD) pathogenesis and plays a crucial regulatory role in myocardial ischemia pathogenesis.

The Qi tonifying and blood stasis eliminating formulation, shen-yuan-dan (SYD), is a commonly used traditional Chinese medicine (TCM), which is used to treat angina pectoris [13]. In our previous study, we used a Waters ultra-high-performance liquid chromatography-tandem mass spectrometer (UPLC-MS/MS) equipped with a HESI-II probe to analyze SYD components and found that the total ion chromatograms of SYD comprise tetrahydropalmatine, harpagoside, salvianic acid A, salvianolic acid B, and tanshinone IIA [14] (Table 1 presents the main chemical components, area of application, and dosage). Our previous studies have shown that SYD can reduce the MI area, myocardial cell injury, and apoptosis in rats with AMI, as well as play a myocardial protective role by upregulating miR-24 expression and interfering with the mitochondrial apoptosis signaling pathway [42]. However, its effect and mechanism of action on hypoxia-induced neonatal rat cardiomyocyte apoptosis in vitro remain unclear. Therefore, the present study attempted to investigate the effect of SYD on hypoxia-induced cardiomyocyte apoptosis and explore whether SYD can reduce ischemic myocardial apoptosis and confer myocardial protection by regulating miR-24/Bim pathway expression.

## 2. Materials and Methods

### 2.1. Reagents and Instruments

TRIzol, Lipofectamin™ 2000 transfection Kit (#11668027), and Power SYBR Green PCR Kit (#4309155) were purchased from Invitrogen, USA. Dual-Luciferase Reporter Assay System and M-MLV reverse transcriptase were obtained from Promega. The mirVana™ qRT-PCR miRNA Detection Kit (#AM1558) was acquired from Life Technologies, USA. The miR-24 mimics and inhibitors were synthesised by the GenePharma Company (Shanghai, China). MTT Detection Kit and TUNEL Apoptosis Detection Kit were procured from Beyotime Institute of Biotechnology (Beijing, China). Lactate dehydrogenase (LDH) and creatine phosphokinase (CPK) detection kits were procured from the Nanjing Jiancheng Bioengineering Institute, China. Annexin V Apoptosis Detection Kit was purchased from BD Biosciences, USA. Bim and GAPDH antibodies were purchased from Cell Signaling. The horseradish peroxidase-labelled secondary antibody was procured from Santa Cruz Biotechnology. The enzyme label instrument, Tecan Infinite M1000, was procured from Switzerland. The ABI 7300 real-time PCR instrument was purchased from Applied Biosystems, USA. The NanoDrop 2000 spectrophotometer was purchased from Thermo, USA. The FacsCalibur flow cytometer was purchased from BD Biosciences, USA. The inverted fluorescence microscope was purchased from Olympus, Japan. The upfront fluorescence microscope was purchased from Leica, Germany.

### 2.2. Preparation of SYD Aqueous Extracts and Pharmacological Serum

SYD comprises eight crude medicinal agents, namely, Salvia miltiorrhiza Bge (15 g), Astragalus membranaceus Bge (12 g), Radix Pilose Asiabell (10 g), Radix Scrophulariae (5 g), Hirudo nipponica (Whitman) (3 g), Lumbricus (5 g), Eupolyphaga sinensis (Walker) (5 g), and Rhizoma Corydalis (5 g) (Table 1). All medicinal herbs were purchased from Beijing Xinglin Pharmaceutical Co. (Beijing, China) and were authenticated by Kechen Mao, a professional herbalist from Beijing TCM Hospital, Capital Medical University. A mixture of all these herbs was soaked in distilled water for 30 min, boiled in 10 volumes of water (v/w) for 1 h, and extracted thrice. The filtered and mixed solution from three decoctions was vacuum concentrated using a rotary evaporator to a final concentration of 1 g/ml (w/v), followed by centrifugation at 3000 rpm for 30 min, and finally stored at −20°C for subsequent experiments. To obtain SYD pharmacological serum, 50 Wistar rats (weight: 220–250 g), purchased from the Institute of Laboratory of Animal Sciences, China Academy of Medical Science (Beijing, China), were divided into two groups; one group was administered SYD (6 mL/kg) and another group (control) was administered saline (6 mL/kg) orally twice daily for five days. One hour after the final administration, rats from each group were anaesthetized. Then, blood was drawn from the abdominal aortic artery, and the blood samples were centrifuged at 3000 rpm for 10 min. Serum from each rat was collected and centrifuged at 1000 rpm for another 10 min. Individual serum samples from the same treatment group were combined in a 4 mL tube, inactivated at 56°C for 30 min, and stored at −20°C before processing. Prior to cellular experiments, sera of both SYD and control groups were diluted to 5% or 10% (v/v) with the DMEM/F12 culture medium.

### 2.3. Myocardial Cell Culture and Modeling

The heart of neonatal SD rats (1–3 days, Beijing Vitong Lihua Experimental Animal Co. Ltd, Certificate No. SCXK (Beijing) 2015-0007) was taken out under sterile conditions. The tissues were subjected to enzymatic digestion to obtain a single-cell suspension and separated using the differential adherent method to obtain purified cardiomyocytes. The cultivated cardiomyocytes were counted, 5 × 10^5^/mL seed plates, and used for the experiments 48 h later. The cardiomyocytes were cultivated for 48 h and then cultured on low-glucose, serum-free DMEM for an additional 12 h to synchronise cell growth. The cardiomyocytes were cultivated for 12, 24, and 48 h in a 1% Air–94% N_2_–5% CO_2_ incubator to establish a hypoxic injury model. Low-glucose DMEM in normal oxygen was used as a control group.

### 2.4. Liposome-Mediated Chemical Synthesis of miR-24 and Its Transfection into Cardiomyocytes

Cardiomyocytes were transfected with the chemically synthesised miR-24 mimic and inhibitor by using Lipofectamine^™^ 2000, according to the manufacturer's instructions.

### 2.5. Experimental Grouping and Cell Processing

A total of five experimental groups were formed: (1) Normoxia; (2) hypoxia; (3) hypoxia + SYD; (4) hypoxia + SYD + miR-24 mimic; and (5) hypoxia + SYD + miR-24 inhibitor. The cardiomyocytes were cultivated for 48 h, and then, the culture medium was changed to low-glucose, serum-free DMEM for another 12 h to synchronise cell growth. Then, the miR-24 mimics and inhibitors were transfected into the neonatal rat cardiomyocytes, cocultivated with SYD-containing serum DMEM for 30 min, and then cultivated in a 1% Air–94% N_2_–5% CO_2_ incubator for 48 h to establish the hypoxic injury model. Myocardial cells with 48 h of ischemia and hypoxia were collected for experimental observations.

### 2.6. Lactic Dehydrogenase and Creatine Phosphokinase Determination

LDH and CPK activities in the supernatant of myocardial cell culture medium were determined colorimetrically, and the effects of SYD serum on the degree of hypoxic myocardial cell injury were observed. Specific methods mentioned in the kit operation instructions were followed.

### 2.7. MTT Assay for Myocardial Cell Viability

Primarily cultivated cardiomyocytes were seeded in 96-well plates at a density of 3-4 × 10^5^/mL (150 *μ*L per hole). After 48 h of hypoxia, 15 *μ*L of MTT solution (5 mg/mL) was added to each well for further incubation at 37°C for 4 h. After four hours, the supernatant was removed, 150 *μ*L dimethylsulfoxide (DMSO) was added to each well, and the plates were shaken for 10 min to allow the crystals to fully dissolve. The absorbance (OD) of the reaction mixture at 490 nm was recorded. The cell survival rate was the percentage of the absorbance value of the experimental group compared with that of the control group.

### 2.8. Detection of Cardiomyocyte Apoptosis through AnnexinV/PI Double Staining and Flow Cytometry

The cells were subjected to trypsin digestion and washed twice with PBS; approximately 3 × 10^5^ cells were collected. The collected cells were suspended in 500 *μ*L Annexin V-binding buffer, followed by staining with 5µL AnnexinV-FITC and 5 *μ*L PI at room temperature in dark for 15 min. Thereafter, the cell apoptosis was detected through flow cytometry. All the experiments were performed in triplicate.

### 2.9. Detection of Cardiomyocyte Apoptosis through TUNEL Staining

After hypoxia was induced, the original culture medium was discarded, and the cells were washed thrice with PBS. Then, the cells were fixed with 4% paraformaldehyde for 60 min. Afterwards, 4% paraformaldehyde was discarded, and the cells were washed with PBS, which was discarded prior to placing the cells in PBS containing 0.1% Triton X-100 to submerge them at the bottom of the board, and then, the cells were placed on ice for 2 min. Then, Triton-PBS was discarded, and the cells were washed twice with PBS. Subsequently, 50 *μ*L TUNEL working solution was added to each well of the plate, and the mixture was incubated at 37°C in dark for 1 h. Five minutes before the endpoint, 50 *μ*L of DAPI solution was added to each well, followed by gentle mixing and incubation in the dark for 5 min. Afterwards, the cells were washed thrice with PBS. The cells were sealed with fluorescent labels and observed through fluorescence microscopy. Five overlapping images (green + purple) were randomly captured from each sample to calculate the proportion of TUNEL-positive cells, which appeared as green fluorescent cells, whereas the remaining cells appeared as purple fluorescent cells.

### 2.10. Quantitative Real-Time PCR

Total RNA from the cardiomyocytes was extracted using TRIzol reagent, according to the manufacturer's instructions, and the 400 ng RNA template was taken from each sample to synthesise cDNA. miR-24 expression was detected using the mirVana™ qRT-PCR miRNA Detection Kit. The expression of Bim was detected using the Power SYBR Green PCR Kit. U6 was used as an internal control of miR-24, and the mRNA level of Bim was normalised to that of GAPDH. The relative expression of mRNA in each sample was calculated using equation 2^−ΔΔCt^. The primers were listed as follows:

  miR-24-F: 5′-GAGCTTGCCAGAGTATCCACG-3′
  miR-24-R: 5′-GCTATTGCCCAAGAGGTCGC-3′
  U6-F: 5′-CTTCACTGGGAAGTTCGGTC-3′
  U6-R: 5′-ACTTCCCTTAGGCATCCCA-3′
  Bim-F: 5′-CTCTTTAAGCCGTTAGCCC-3′
  Bim-R: 5′-GATCCTTGGACCGTCCTGTA-3′
  GAPDH-F: 5′-CTGCGGATTT GGACAGTTCC-3′
  GAPDH-R: 5′-ACTTCCTTCAGAGATCCCAT-3′

### 2.11. Western Blot Analysis

The cells were subjected to trypsin digestion and then collected. RIPA lysate was added, lysed on ice for 30 min, centrifuged at 12000 rpm for 4 min at 4°C, and the supernatant was collected. Protein concentration was determined using the BCA method. Subsequently, 50 *μ*g of protein lysates was separated through 10% SDS-PAGE and were then transferred to the PVDF membrane. After blocking the membrane with 5% skimmed milk at room temperature for 1 h, GDPDH or primary antibodies were added, followed by overnight incubation at 4°C. After washing the membrane thrice with TBST buffer solution, horseradish peroxidase-labelled secondary antibodies were added, and the mixture was incubated at room temperature for 1 h. The films were washed thrice with TBST and subjected to the ECL chemiluminescence assay; the images were captured using the Image-Pro plus 6.0 image analysis system.

### 2.12. Luciferase Reporter Assays

The full-length sequence amplification product of Bim gene 3′ UTR, which contained the predicted miR-24 targeting regions, was inserted into a PmirGLO expression vector (Bim-wt), and targeted mutation (Bim-mut) was performed for the target binding site of miR-24 and Bim gene predicted using a software (TargetScan 3.0). Then, Lipofectamine™ 2000 was used to cotransfect the reporter plasmid and miR-24 mimic or its negative control (miR-24 mimic-NC) into HEK293T cells. Bim-wt and Bim-mut transfected cells were divided into three groups, namely, blank control, miR-24 mimic, and mimic negative control, with three duplicate wells in each group. After a 48 h incubation period, luciferase activity was detected using the Tecan M1000 multifunctional enzyme labelling instrument, according to the instructions provided in the double luciferase activity detection kit. Relative luciferase activity was calculated using the following formula: relative luciferase activity = firefly luciferase activity/sea kidney luciferase activity. The experiment was repeated three times.

## 3. Statistical Analysis

SPSS 15.0 software was used for data analyses. All data are presented as mean ± SEM (standard error of mean) from at least three independent experiments. Differences between the groups were determined using the Student's *t*-test and one-way ANOVA. Statistical significance was considered at a *P* value of <0.05.

## 4. Results

### 4.1. Effect of SYD on Morphology, Injury Degree, and Viability of the Hypoxia-Induced Cardiomyocyte

**Figure 1:**
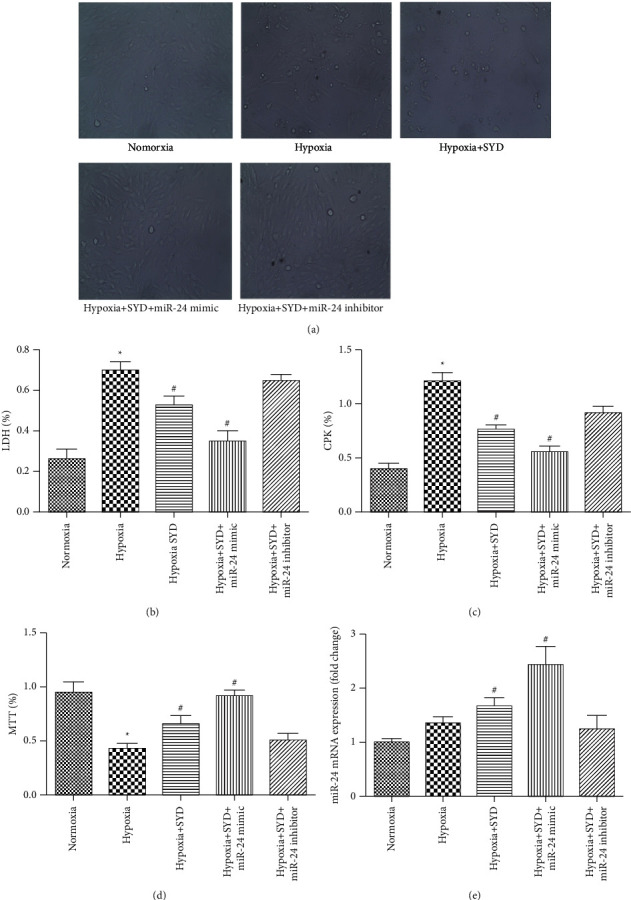
Effects of SYD on hypoxia-induced cardiomyocyte morphology, cell injury, cell viability, and miR-24 expression. (a) After 48 hours of hypoxia, the morphology of myocardial cells changed from spindle to round or quasi-round. (b, c) SYD could reduce LDH and CPK activity of hypoxia myocardial cells. (d) Cardiomyocytes viability was examined by MTT assay. (e) The expression of miR-24 was detected by qRT-PCR. Data are expressed as the mean ± SEM (*n* = 3). ^★^*P* < 0.05 versus Normoxia group; ^#^*P* < 0.05 versus Hypoxia group.

Cardiomyocyte morphology was found to change 48 h after hypoxia (Figure 1(a)); the morphology changed mainly from spindle to round or quasi-round. LDH and CPK activities increased (Figures 1(b) and 1(c)), cell survival rate decreased (Figure 1(d)), and miR-24 expression increased by 35% (Figure 1(e)). These results indicated that hypoxia induces myocardial cell injury and that miR-24 may play a role in hypoxia-induced myocardial cell injury.

Then, we observed the effect of SYD serum on hypoxia-induced myocardial cell injury and found that SYD could reduce the LDH and CPK activities of the hypoxic myocardial cells (Figures 1(b) and 1(c)) and increase the cell survival rate (Figure 1(d)). Interestingly, miR-24 level in the hypoxia + SYD group was upregulated by 67%, as compared to that of the control (Figure 1(e)). This result suggested that SYD can alleviate hypoxic myocardial cell injury, and SYD treatment can lead to upregulation of miR-24.

To determine whether miR-24 plays a role in SYD-mediated protection to hypoxic myocardial cells, we transfected the miR-24 mimic and inhibitor into the cardiomyocytes and cocultivated with SYD-containing serum. The MTT assay showed that the viability of myocardial cells was further improved (Figure 1(d)) and the activities of LDH and CPK were further decreased (Figures 1(b) and 1(c)) in the miR-24 mimic transfection group. However, after miR-24 inhibitor transfection, the viability of cells was slightly decreased (Figure 1(d)), and the activities of LDH and CPK were increased (Figures 1(b) and 1(c)) compared with those in the hypoxia + SYD group. These results indicated that upregulation of miR-24 can significantly enhance the protective effect of SYD in hypoxia-induced cardiomyocyte.

### 4.2. Effect of SYD on Apoptosis of Hypoxic-Induced Cardiomyocytes

**Figure 2:**
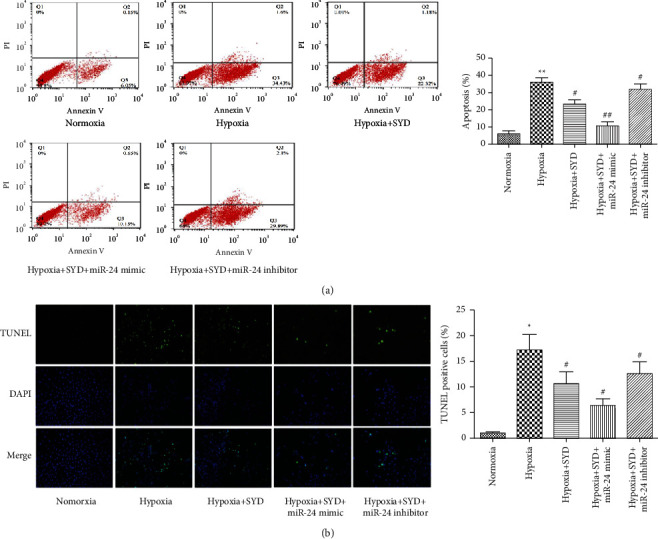
Effects of SYD on hypoxia-induced cardiomyocyte apoptosis. (a) Detection of hypoxia-induced cardiomyocyte apoptosis by flow cytometry. (b) Detection of hypoxia-induced cardiomyocyte apoptosis by TUNEL staining. Data are expressed as the mean ± SEM (*n* = 3). ^★^*P* < 0.05 and ^★★^*P* < 0.01 versus Normoxia group; ^#^*P* < 0.05 and ^##^*P* < 0.01 versus Hypoxia group.

To determine the effect of SYD and the role of miR-24 in hypoxia-induced cardiomyocyte apoptosis, we used flow cytometry and TUNEL methods to detect apoptosis. Flow cytometry indicated that after 48 h of hypoxia, the apoptosis rate of the cardiomyocytes was significantly increased, and SYD could effectively reduce the apoptosis rate of the cardiomyocytes. The apoptosis rate of the miR-24 mimic group was further reduced, whereas that of the miR-24 inhibitor group was slightly increased compared with that of the hypoxia + SYD group (Figure 2(a)). Similar results were obtained through TUNEL staining. After transfection with the miR-24 mimic, the proportion of TUNEL-positive cells was significantly reduced, while transfection with the miR-24 inhibitor displayed the opposite effect (Figure 2(b)).

### 4.3. Effects of SYD on mRNA and Protein Expression of Bim in the Hypoxic-Induced Cardiomyocytes

**Figure 3:**
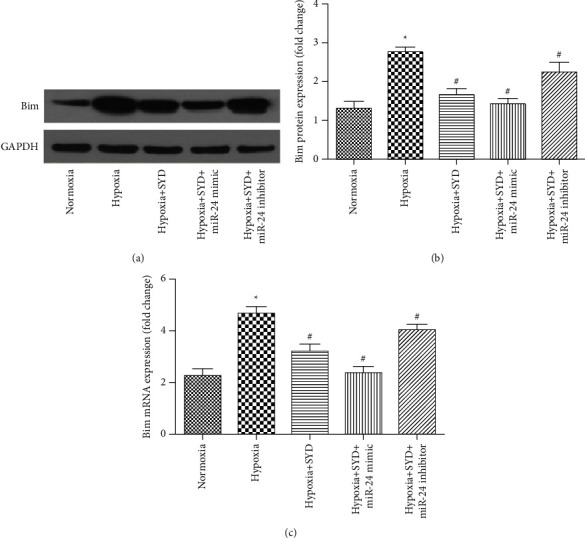
Effect of SYD on the expression of Bim protein and mRNA in hypoxia-induced cardiomyocyte. (a) The expression of Bim protein was analyzed by western blotting. (b) SYD can decrease the expression of Bim protein in hypoxia-induced cardiomyocyte. (c) SYD can decrease the expression of Bim mRNA in hypoxia-induced cardiomyocyte. Data are expressed as the mean ± SEM (*n* = 3). ^★^*P* < 0.05 versus Normoxia group; ^#^*P* < 0.05 versus Hypoxia group.

Western blot and qRT-PCR analysis showed that, after 48 hours of hypoxia, the Bim protein and mRNA expression were significantly increased and SYD could decrease the protein and mRNA expression of Bim. Compared with the hypoxia + SYD group, Bim protein and mRNA expression in the miR-24 mimic group were further decreased (Figures 3(a)–3(c)). These results suggest that SYD can improve hypoxia-induced cardiomyocyte apoptosis by upregulating miR-24 expression and inhibiting the Bim expression.

### 4.4. Luciferase Report Assay Showed Bim Was the Target Gene of miR-24

**Figure 4:**
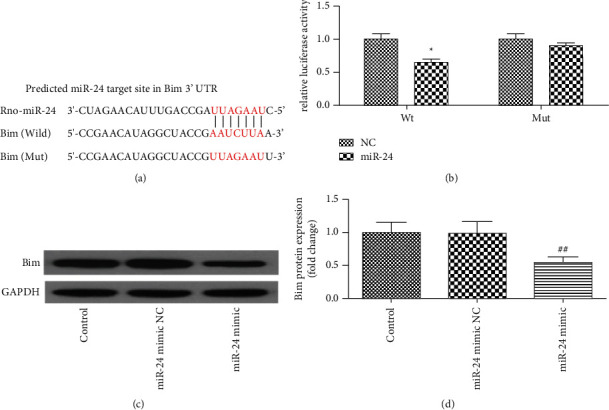
Bim is the direct target gene of miR-24. (a) Schematic description of the base-pairing interaction between miR-24 and Bim mRNA. (b) Luciferase reporter assay was performed in neonatal rat cardiomyocytes that were cotransfected with miR-24 mimics or NC-mimics together with reporter vectors containing Bim 3′UTR or mutated Bim 3′UTR. Relative luciferase activities were presented. (c, d) Western blot analysis showed that the expression of Bim protein was significantly decreased after transfection with miR-24 mimic in neonatal rat cardiomyocytes. ^★^*P* < 0.05 versus NC group; ^##^*P* < 0.01 versus miR-24 mimic NC group.

To elucidate the specific mechanism of action of miR-24, we predicted the target gene of miR-24 through TargetScan and identified Bim as the target gene of miR-24. The pmirGLO-report plasmid of wild-type and mutated 3′ UTR of Bim, which contained the binding sites of miR-24, was cloned downstream of the luciferase reporter gene vector. Additionally, cotransfection of the miR-24 mimic and the plasmid with the Bim-wt 3′ UTR inhibited the luciferase activity of the Bim gene. However, cotransfection of the miR-24 mimic and the plasmid with the Bim-mut 3′ UTR demonstrated no significant effect on the luciferase activity of the Bim gene (Figures 4(a) and 4(b)), indicating that miR-24 regulates its expression by directly targeting the 3′ UTR of the Bim gene. Meanwhile, western blot analysis showed that Bim protein expression was significantly decreased after transfection with miR-24 in the neonatal rat cardiomyocytes (Figures 4(c) and 4(d)). These results indicated that miR-24 can inhibit Bim expression by binding to the 3′ UTR of Bim.

## 5. Discussion

The present study revealed that SYD treatment can effectively alleviate hypoxia-induced neonatal rat cardiomyocyte injury, improve cell viability, inhibit cardiomyocyte apoptosis, and regulate the expression of apoptotic-related factors of the mitochondrial cell apoptosis pathway. Furthermore, our results confirmed that Bim is the direct target gene of miR-24 and that the upregulation of miR-24 expression in cardiomyocytes can significantly inhibit the target gene Bim and enhance the protective effect of SYD, thereby reducing myocardial cell apoptosis. Finally, our results confirmed that SYD can play a protective role in reducing hypoxia-induced cardiomyocyte apoptosis by upregulating miR-24 expression and inhibiting the expression of its target gene Bim.

Apoptosis is a key pathological mechanism following AMI and is mediated by the mitochondrial pathway, in which the interaction among the Bcl-2 protein family members plays a key role in regulating apoptosis [3]. When hypoxia or ischemia occurs, pro-apoptotic proteins (Bad and Bax) are translocated to the mitochondria, which induce the downregulation or degradation of Bcl-2 and Bcl-xL, eventually increasing the release of mitochondrial cytochrome C (Cyt-C). When Cyt-C is released from the mitochondria, it changes conformation with APaf-1 (apoptosis protein-activated factor) in the presence of ATP/dATP and forms a large structure called an apoptosome. Simultaneously, the apoptosome attracts Caspase-9 precursors, and Caspase-9 is activated through trans-catalysis after polymerisation with the apoptosome structure. The activated Caspase-9 then acts on the downstream Caspase-3, Caspase-6, and Caspase-7, thereby activating the caspase-cascade and causing apoptosis. For terminally differentiated cardiomyocytes lacking the regenerative capacity, apoptosis of myocardial cells after MI should be decreased for promoting the recovery of cardiac function and delaying the incidence of heart failure after MI. The results of our study demonstrated that after 48 h of hypoxia, the LDH and CPK activities were obviously increased, the cell viability was decreased, and the myocardial cell apoptosis was significantly increased. SYD can effectively reduce the activities of LDH and CPK, improve myocardial cell viability, and inhibit cell apoptosis in hypoxia-induced cardiomyocytes. Our study also indicated that SYD intervention could decrease the gene and protein expression of pro-apoptotic factor Bim. These results confirm that SYD can play a protective role in myocardium by reducing hypoxia-induced apoptosis of the cardiomyocytes.

Studies have shown that miR-24, which is highly expressed in the mammalian myocardium and other tissues, can participate in the pathological process of various cardiovascular diseases such as myocardial hypertrophy, apoptosis, myocardial fibrosis, and cardiac remodeling by regulating the translation and expression of its target genes [43–46]. Li et al. [47] found that miR-24, which is highly expressed in heart failure and myocardial hypertrophy models, can suppress the stress response of cardiomyocytes by inhibiting the expression of its target gene JP2, whereas the downregulation of miR-24 was found to stabilize JP2 protein expression, thereby stabilizing the electrophysiological process and stress response of cardiomyocytes. Qian et al. [10] found that miR-24 can regulate the apoptosis of myocardial cells after MI by targeting the expression of Bim pro-apoptotic genes. After MI, miR-24 expression in the myocardial tissues of the infarction area is significantly reduced, and upregulation of miR-24 expression can reduce the apoptosis of myocardial cells in the infarction area. All the aforementioned studies suggest that miR-24 may be a protective factor involved in the pathology of myocardial cell injury and repair after MI. In this study, we first found that hypoxia causes myocardial cell injury and upregulation of miR-24. SYD treatment significantly improved myocardial cell injury induced by hypoxia. Interestingly, the expression level of miR-24 was further increased in response to SYD treatment in hypoxic surroundings. To investigate the role of miR-24 in myocardial cell injury, miR-24 mimics or inhibitors were transfected to hypoxia + SYD-treated myocardial cells. The result showed that transfection of miR-24 mimics further improved myocardial cell injury, while transfection of miR-24 inhibitors enhanced myocardial cell injury. Therefore, miR-24 plays a protective role in hypoxia-mediated myocardial cell injury.

According to Chinese medical theory, AMI falls within the scope of “True Heart Pain,” and the “Qi deficiency and blood stasis syndrome” is the most common syndrome in TCM clinical practice. Tonifying Qi and expelling stasis are highly significant in the treatment of CHD. SYD is a TCM herbal compound preparation that invigorates Qi and removes blood stasis. This preparation comprises Astralgus membranaceus Bge, Radix Pilose Asiabell, Radix Scrophulariae, Salvia miltiorrhiza Bge, Lumbricus, Eupolyphaga sinensis, Hirudo nipponica, and Rhizoma Corydalis. Studies have reported that SYD can effectively reduce the plasma LDH and CK-MB levels in rats with ischemia/reperfusion injury, prevent lipid peroxidation injury, inhibit inflammatory response, and slow down the expansion of infarct after MI in rats. SYD can also increase the activity of myocardial cells induced by hypoxia/reoxygenation [13]. Our previous in vivo experiments also indicated that SYD can effectively reduce the myocardial apoptosis rate in MI rats, increase miR-24 expression, and play an anti-apoptosis role by inhibiting the expressions of the target gene Bim and downstream pro-apoptotic factors [42]. In this study, we found that SYD can alleviate hypoxia-induced injury and reduce apoptosis of neonatal rat myocardial cells. In addition, we found that the inhibitory effect of SYD on apoptosis was further enhanced after transfection with an miR-24 mimic, whereas this protective effect was significantly weakened after transfection with an miR-24 inhibitor. Based on these results, we can conclude that the mechanism for the decreased hypoxia-induced apoptosis of cardiomyocytes caused by SYD may be related to the upregulation of miR-24 expression and the inhibition of the expression of its target gene Bim.

## 6. Conclusion

SYD can alleviate hypoxia-induced neonatal rat cardiomyocyte injury, decrease LDH and CPK activities, increase cell viability, and reduce cell apoptosis. Meanwhile, our results confirmed that SYD could upregulate miR-24 expression and downregulate mitochondrial apoptotic pathway pro-apoptotic factor Bim expression. Bioinformatics analysis and luciferase reporter assays showed that Bim is the direct target gene of miR-24. Moreover, upregulation of miR-24 expression could significantly inhibit the expression of the target gene Bim, thereby enhancing the effect of SYD and decreasing myocardial cell apoptosis in the neonatal rat cardiomyocytes. Our results highlight that SYD treatment can reduce hypoxia-induced myocardial apoptosis by upregulating miR-24 expression. This study offers novel insights into the molecular mechanism underlying the therapeutic potential of SYD in promoting the recovery of myocardial function and delaying heart failure incidence.

## Figures and Tables

**Table 1:** Composition of shen-yuan-dan.

English name	Latin name	Chinese name	Place of production (province)	Collecting time (season)	Part used	Amount used (g)	TCM efficacy	Pharmacological activity
Salvia miltiorrhiza Bge	Radix Salviae	Danshen	Hubei, Henan	Spring and autumn	Root and rootstock	15	Activating blood and removing stasis; inducing menstruation to relieve menalgia; eliminating the heart-fire; cooling-blood	Inhibiting increase of blood serum level of triglycerides [15], reversing renal injury induced [16], reversing cerebral ischemia-reperfusion injury [17], relieving myocardial ischemia [18], ameliorating diabetic vascular injury [19]
Astragalus membranaceus Bge	Astragalus propinquus	Huangqi	Shandong	Autumn	Root	12	Invigorating Qi for strengthening superficies; diuresis; promoting granulation	Inhibiting myocardial ischemia-reperfusion injury, myocardial hypertrophy, vascular endothelial dysfunction, coronary heart disease, atherosclerosis, cardiac fibrosis, viral myocarditis, and diabetes mellitus [20], exerting antioxidant activity [21]
Radix Pilose Asiabell	Codonopsis Radix	Dangshen	Gansu	Autumn	Root	10	Tonifying middle-Jiao and Qi; promote the production of body fluid; strengthening spleen; and tonifying lung	Preventing hypoxia-induced platelet activation and resultant procoagulant state [22], promoting mouse immune function [23], inhibiting the development of diet-induced obesity and hyperlipidemia [24], and relieving myocardial ischemia-reperfusion injury [25]
Radix Scrophulariae	Scrophularia ningpoensis	Xuanshen	Hebei	Winter	Root	5	Removing pathogenic heat from blood; nourishing yin for lowering fire; clearing toxic material, dispersing mass	Reversing heart and cerebral ischemia/reperfusion injury [26], relieving diabetic nephropathy [27], inhibiting the ventricular remodeling induced by hypertension [28], attenuating arteriosclerosis [29]
Hirudo nipponica (Whitman)	Whitmania pigra Whitman	Shuizhi	Shandong	Summer and autumn	All parts of the Hirudo	3	Broken blood stasis, pursue action of silt, stimulate the menstrual flow	Promoting blood circulation and removing stasis [30], relieving atherosclerosis [31], alleviating thrombus burden [32], improving blood hyperviscosity and related metabolic disorders [33]
Lumbricus	Pheretima	Dilong	North China	Summer and autumn	All parts	5	Dredging collaterals, relieving cough and asthma, clearing heat, diuretic	Exerting anti-thrombus [34], reversing mitochondrial injury and pro-fibrotic events [35], protecting brain microvascular endothelial cells after oxygen-glucose deprivation/reperfusion [36]
Eupolyphaga sinensis (Walker)	Eupolyphaga seu Steleophaga	Tubiechong	North China	Summer and autumn	All parts	5	Broken blood stasis, reunion of fractured tendons and bones	Relieving atherosclerosis [37], exerting antiangiogenesis [38]
Rhizoma Corydalis	Corydalis Rhizoma	Yanhusuo	Zhejiang; Jiangsu	Summer	Tubers	5	Removing blood stasis; regulating Qi; and relieve pain	Degrading the blood clot and delaying the plasma recalcification time [39], inhibiting inflammation, myocardial fibrosis, and platelet aggregation [40], improving the hypercoagulable state [41]

## Data Availability

The datasets used and/or analyzed during the current study are available from the corresponding author on reasonable request.

## Abbreviations

SYD: Shen-yuan-dan
AMI: Acute myocardial infarction
miRNAs: MicroRNAs
TCM: Traditional Chinese medicine
UPLC-MS/MS: Ultra-high-performance liquid chromatography-tandem mass spectrometry
DMSO: Dimethylsulfoxide
qRT-PCR: Quantitative real-time PCR
LDH: Lactic dehydrogenase
CPK: Creatine phosphokinase.
